# Medication Adherence and Lifestyle Changes Among Treated Patients with Arterial Hypertension in Family Medicine in Croatia According to the EUROASPIRE V Study: A Cross-Sectional Study

**DOI:** 10.3390/jcm15145376

**Published:** 2026-07-09

**Authors:** Renata Romic, Venija Cerovecki, Ino Kermc, Zlata Ozvacic Adzic, Goranka Petricek, Miroslav Hanzevacki, Natasa Buljan, Pero Hrabac, Jure Samardzic, Zeljko Reiner, Davor Milicic

**Affiliations:** 1 Health Centre Zagreb—Centar, 10000 Zagreb, Croatia; renata.brtan@gmail.com (R.R.); ino.kermc@mef.hr (I.K.); zlata.ozvacic@mef.hr (Z.O.A.); 2 Department of Family Medicine, University of Zagreb School of Medicine, 10000 Zagreb, Croatia; goranka.petricek@mef.hr (G.P.); miroslav.hanzevacki@mef.hr (M.H.); 3 Health Centre Zagreb—West, 10000 Zagreb, Croatia; 4 Family Medicine Private Practice Nataša Buljan, 10000 Zagreb, Croatia; dr.n.buljan@gmail.com; 5 Department of Medical Statistics, Epidemiology and Medical Informatics, University of Zagreb School of Medicine, 10000 Zagreb, Croatia; pero.hrabac@mef.hr; 6 Department of Cardiovascular Diseases, University Hospital Centre Zagreb, 10000 Zagreb, Croatia; jure.samardzic@gmail.com (J.S.); davormilicic01@gmail.com (D.M.); 7 Department of Internal Medicine, University of Zagreb School of Medicine, 10000 Zagreb, Croatia; 8 Department of Metabolic Diseases, University Hospital Centre Zagreb, 10000 Zagreb, Croatia; zeljko.reiner@kbc-zagreb.hr

**Keywords:** arterial hypertension, adherence, lifestyle characteristics, primary care

## Abstract

**Introduction:** Arterial hypertension (AH) is one of the leading public health issues worldwide, and patients’ adherence to recommended treatment is essential for successful regulation of blood pressure (BP), disease control, and prevention of complications. **Aim:** The aim of this study was to evaluate the adherence of patients with hypertension to medication and lifestyle changes in Croatia, based on the primary care arm of the EUROASPIRE V survey. **Methods:** A cross-sectional study was conducted in nine family medicine practices in the city of Zagreb, following the protocol of the EUROASPIRE V primary care study. Out of the total Croatian cohort of 203 participants, data on 156 participants with AH were included in this study. The study used components of the EUROASPIRE V questionnaire on self-assessment of adherence to medications, existing lifestyle characteristics and self-reported lifestyle changes (smoking, salt intake, and physical activity, respectively). **Results:** A total of 156 participants were included, with almost even distribution of men (*n* = 77, 49.4%) and women (*n* = 79, 50.6%); median age was 65 (57, 72). A self-reported medication adherence to antihypertensive medication of 100% (all of the time) was reported by 56.9% patients. According to the lifestyle change questionnaire, the highest adherence was concerning reduction in salt intake (74.4%), while smoking was reduced in 24.1% of advised participants with AH, 13.8% had quit smoking, and increased physical activity was reported by 24.4% patients. **Conclusions:** The results of the primary care arm of EUROASPIRE V study in Croatia on treated patients with AH indicate suboptimal BP control in a significant proportion of patients (55.1%) and suboptimal lifestyle change adherence. Achieving target BP and optimal disease control requires additional efforts to foster patients’ adherence to prescribed medications and lifestyle recommendations.

## 1. Introduction

Elevated BP or AH (BP ≥ 140/90 mmHg) is a silent disease that may be asymptomatic for years until target-organ damage develops [1]. As a global epidemic, AH is a leading risk factor for worldwide mortality and morbidity, affecting an estimated 1.4 billion adults aged 30–79 years, according to the World Health Organization (WHO) [1].

In Croatia, cardiovascular diseases remain the primary cause of mortality [2]. According to Eurostat data from 2019, Croatia had the highest prevalence of elevated BP among the EU countries, affecting 37% of individuals aged 15 years and older [3]. Current national data (EHUH-2 study) indicate that AH prevalence in Croatia reached 51.7%, meaning that more than 1.6 million citizens have AH [4]. Consequently, these data support the conclusion that AH remains a major and growing public health issue in Croatia as well.

The data indicate that nearly 50% of people with AH are unaware of their elevated BP and even among individuals who are aware of their AH, a substantial proportion do not take adequate measures, such as regularly taking prescribed medications or adopting healthier lifestyle habits [4,5,6,7,8]. Medication adherence is defined as taking medications as prescribed to achieve a therapeutic outcome [9]. Contemporary evidence indicates that non-adherence to antihypertensive therapy affects approximately one-third to one-half of patients with AH, making it one of the principal causes of inadequate BP control worldwide [10]. Recent systematic reviews have confirmed that poor adherence is consistently associated with uncontrolled BP, increased cardiovascular morbidity and mortality, and higher healthcare utilization [11]. Reflecting the importance of this issue, both the European Society of Hypertension (ESH) and the European Society of Cardiology (ESC) recognize medication adherence as a keystone of effective hypertension management [5,6].

The most significant and well-established lifestyle measures that have been shown to reduce premature cardiovascular-related morbidity and mortality include achieving and maintaining optimal body weight, adhering to the DASH dietary pattern, reducing sodium intake, increasing potassium intake, implementing regular physical activity and structured exercise programmes, and smoking cessation. In addition, certain dietary components such as intake of polyphenols, coffee and tea, as well as various stress-reduction techniques (e.g., mindfulness techniques, autogenic training), are also associated with improved BP regulation [5,6]. Maintaining long-term patient adherence presents a significant challenge in the implementation of both pharmacological and non-pharmacological measures. Poor adherence to medication and lifestyle changes is a result of the interplay of factors related to the disease and the patient’s disease perception, the type of therapy, socioeconomic circumstances and the specific of the national health system. Improving BP regulation and achieving target values require intensified efforts from medical professionals aimed at strengthening medication adherence and promoting healthy lifestyle habits [6,7,12,13].

The EUROASPIRE programme, led by the ESC, evaluates the implementation of cardiovascular prevention guidelines in everyday clinical practice across Europe. The primary care arm of EUROASPIRE V focused on individuals at high cardiovascular risk receiving treatment for hypertension, dyslipidaemia, and/or diabetes. Because poor adherence to antihypertensive treatment and healthy lifestyle recommendations remains a major challenge in achieving optimal BP control and reducing cardiovascular risk, understanding adherence patterns in primary care is highly relevant. Therefore, this study aimed to evaluate the adherence of patients with AH to antihypertensive medication and lifestyle recommendations, using data from the primary care arm of the EUROASPIRE V survey in Croatia. The results may help identify opportunities to improve cardiovascular prevention and patient care.

## 2. Materials and Methods

### 2.1. Study Population

This study was conducted as part of the EUROASPIRE V primary care arm coordinated by the European Society of Cardiology EURObservational Research Programme (EORP), involving 16 European countries, including Croatia. To ensure comparability across countries, standardized procedures were applied throughout the study. According to the EUROASPIRE V protocol, countries with fewer than 5 million inhabitants were allowed to recruit participants from a single geographic area with a population exceeding 500,000. Accordingly, Zagreb was selected as the study area, and nine family medicine practices participated, contributing equally to patient recruitment.

A cross-sectional study design with retrospective identification of eligible participants was employed. The study population comprised adults aged 18–79 years without established atherosclerotic cardiovascular disease (ASCVD) who had been receiving pharmacological treatment for at least one major cardiovascular risk factor (AH, dyslipidaemia, or hyperglycaemia) for at least six months before enrolment. Eligible participants were identified through medical records and invited to attend a study visit. Smoking status was based on self-report.

Sociodemographic and clinical data, including cardiovascular risk factors, medication use, and lifestyle characteristics, were collected during study visits using the standardized EUROASPIRE V questionnaire and protocol. Medication adherence and lifestyle characteristics were assessed by face-to-face interview, while BP was measured according to standardized EUROASPIRE V procedures. BP was measured twice on the right upper arm in the sitting position using an automatic digital sphyngomanometer (Omron Comfort M6, OMRON corporation, Kyoto, Japan). Raised BP was defined as systolic BP ≥140 mmHg and/or diastolic BP ≥90 mmHg.

### 2.2. Adherence Assessment

Medication adherence was assessed using the medication adherence questionnaire included in the standardized EUROASPIRE V survey. Participants were asked to estimate how often they took their prescribed antihypertensive medication according to the physician’s instructions, with responses expressed as percentages (0–100%). Adherence was evaluated based on the self-reported proportion of time participants reported taking their medication as prescribed.

Lifestyle characteristics (salt intake and dietary changes, smoking habits, and physical activity) were also assessed using self-reported questions included in the standardized EUROASPIRE V questionnaire.

The sample size was predefined by the EUROASPIRE V study protocol, which specified a target sample of approximately 200 eligible participants for Croatia. Out of the total Croatian cohort of 203 participants, 156 individuals with AH were included in the present analysis. Participants who did not report current use of prescribed antihypertensive medication (*n* = 47) were excluded, as assessment of medication adherence was one of the primary study outcomes. No additional exclusion criteria were applied.

### 2.3. Statistical Analysis

Statistical analysis was performed using Jamovi software (The Jamovi Project, Version 2.7.34.0), and all data were entered into a secure electronic database. Continuous variables are presented as median ± interquartile range, while categorical variables are summarized as counts and percentages. The normality of the distribution of continuous variables was assessed using the Shapiro–Wilk test. Descriptive statistics were used to estimate the prevalence of antihypertensive medication class, medication class adherence, lifestyle characteristics, and self-reported lifestyle changes (smoking, salt intake, and physical activity). Between-group comparisons according to sex and age categories were conducted using Fisher’s exact test for categorical variables, and a *p*-value < 0.05 was considered statistically significant.

Adherence to antihypertensive therapy was modelled as a binary outcome (adherent versus non-adherent) using hierarchical binary logistic regression. Predictor blocks were entered sequentially, ordered from stable background characteristics toward more proximal behavioural and treatment-related factors, with each block’s incremental contribution assessed through the likelihood-ratio χ^2^ test and the change in pseudo-R^2^.

The first block established patient context and served as the adjustment set for subsequent blocks: age, sex, education, employment status, marital status, major life stresses in the previous year, and alcohol consumption; the SCORE2 cardiovascular risk estimate and body mass index; and five risk-perception items covering the wish to know one’s own CV risk, worry about developing disease, worry about finding out one’s risk, and the perceived likelihood and relative level of that risk.

The second block added medication regimen and psychological factors: whether any prescribed medication was a fixed-dose combination of two or more drugs in a single tablet, and the anxiety and depression subscale scores of the Hospital Anxiety and Depression Scale (HADS), which are both established correlates of poorer adherence.

The third block captured smoking status and history—ever smoked, total years smoked and current smoking status.

The fourth block covered smoking-cessation advice and behaviour over the preceding three years: verbal advice to stop smoking, written cessation materials, and the cessation outcomes of abstinence and reduction.

The fifth block addressed dietary self-management of salt intake, comprising the corresponding advice and behaviour items.

The sixth and final block introduced BP control and the pharmacological regimen: a special doctor-prescribed diet to lower BP, self-reported actual and target BP values, uncontrolled BP (>140/90 mmHg), and the systolic and diastolic readings, plus indicators of treatment with an ACE inhibitor, beta-blocker, angiotensin receptor blocker, calcium-channel blocker, or diuretic. Because control may be partly a consequence of adherence rather than solely a determinant, these associations were interpreted with caution.

## 3. Results

**Figure 1:**
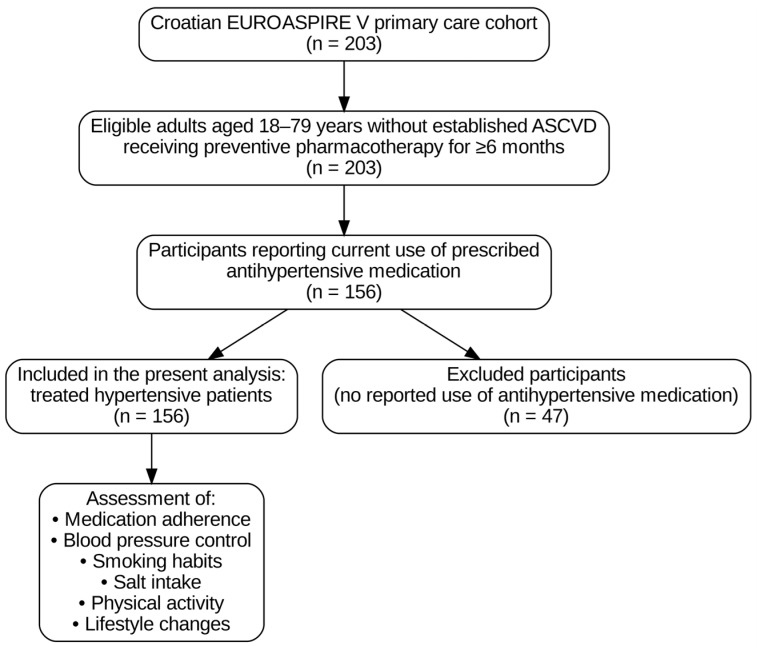
Flowchart of participant selection and inclusion in the present study. ASCVD, atherosclerotic cardiovascular disease.

The participant selection process is presented in Figure 1.

Basic sociodemographic, economic and clinical characteristics of the study population are presented in Table S1 (see Supplementary Materials): a total of 156 participants were included, with almost even distribution of men (*n* = 77, 49.4%) and women (*n* = 79, 50.6%). Median age was 65 (57, 72). Those <60 years represented 34.6% (*n* = 54) of the sample, and those ≥60 years accounted for 65.4% (*n* = 102).

Patients who responded to the question on adherence to prescribed antihypertensive medication therapy were stratified into two groups: those with BP < 140/90 mmHg and those with BP ≥ 140/90 mmHg (Table 1). The proportion of participants with controlled BP was 44.9% (N = 70). The majority of participants in both groups reported a high level of adherence to prescribed antihypertensive therapy, with 56.9% reporting complete medication adherence (all of the time—100%). Participants with regulated BP (<140/90 mmHg) more frequently reported taking therapy in full accordance with instructions. However, the majority of participants in the group with unregulated BP also reported taking antihypertensive medication in full or almost full accordance with instructions (100% or 90% of the time).

Antihypertensive drug classes, the proportion of patients reporting 100% self-assessed adherence within each drug class, and BP control within each drug class are presented in Table 2. Across all antihypertensive drug classes, 100% adherence was reported at similar levels (54.5–59.5%).

The majority of participants reported not smoking, while 18.6% reported that they are current smokers. Regarding smoking cessation, 86.2% of current smokers reported that they have received medical advice to quit smoking in the past three years. Of those, the majority continued smoking, whereas only 13.8% reported successful cessation. A total of 13.8% of the hypertensive smokers claimed that they did not receive such advice, and one participant was unsure whether he had received such recommendation or not. Twenty-nine participants answered the question about reducing smoking in the last 3 years but only 24.1% reported that they have reduced it.

A total of 156 patients responded to the question regarding medical advice on reduction in salt intake during the past three years. The majority reported that they have received such advice, while 19.2% stated that they had not received it. Regarding their response to advice, 74.4% participants answered affirmatively and they had reduced their salt intake in the same period.

Almost half of participants (41.7%) have received physician’s advice to increase physical activity while 24.4% indicated that they had actually increased their activity during the past 3 years. Limitation on physical activity due to illness was reported by 51.9% of participants. The largest proportion of participants, 60.9%, reported engaging in only light physical activity outside work, and 12.8% did not engage in any activity at all. Regarding vigorous physical activity, the majority of participants (81.1%) reported no such activity (lasting longer than 15 min) during a seven-day period. In terms of moderate activity (e.g., brisk walking or light cycling), 10.3% of participants reported engaging in it at least twice a week, while 9.7% reported doing so five times per week. Light physical activity, such as walking, was reported by only 19.3% of participants at a frequency of at least five times per week and by 26.2% on a daily basis. Regarding vigorous activities leading to sweating and increased heart rate, over half of the participants (53.8%) stated that they have engaged in such exercise rarely or never. Regular physical activity of at least 30 min five times per week was reported by 28.2% of participants. When asked about regular exercise according to defined criteria (planned physical activity performed to increase physical fitness, 3 to 5 times per week, for 20–60 min, through, e.g., brisk walking, jogging, cycling, or swimming), 32.1% of respondents reported neither exercising nor having plans to start, while 29.5% intended to begin within the next six months, and only 11.5% of participants reported regular exercise in accordance with these criteria for a period exceeding six months. Table 3 summarizes key results regarding lifestyle characteristics and self-reported lifestyle changes in patients with AH.

Adherence to antihypertensive therapy was prevalent, with approximately 86% of patients classified as adherent. Because of this class imbalance, several categorical predictors in the binary logistic regression analysis (described in detail in Section 2.3) could not be reliably estimated owing to complete or quasi-complete separation. These predictors were therefore treated as artefacts and are not reported below. In the socio-demographic and clinical block, the addition of employment status (ΔΧ^2^ = 13.36, *p* = 0.038) and SCORE2 (ΔΧ^2^ = 5.30, *p* = 0.021) significantly improved model fit. Self-employment was associated with lower odds of adherence relative to the reference category (OR = 0.16, 95% CI 0.03–0.75, *p* = 0.020). Higher SCORE2 was associated with lower odds of adherence (OR = 0.85 per unit, 95% CI 0.76–0.95, *p* = 0.004), and older age with higher odds of adherence (OR = 1.12 per year, 95% CI 1.04–1.22, *p* = 0.005). Higher HADS anxiety score was the strongest predictor, significantly improving fit (ΔΧ^2^ = 21.59, *p* < 0.001) and associated with lower odds of adherence (OR = 0.73 per point, 95% CI 0.62–0.85, *p* < 0.001). Current smoking was associated with lower odds of adherence (OR = 0.28, 95% CI 0.10–0.75, *p* = 0.012). In the smoking-cessation advice sub-analysis, receipt of written stop-smoking materials was associated with lower odds of adherence (OR = 0.13, 95% CI 0.03–0.63, *p* = 0.011). In the BP-control block, not being on a special prescribed diet was associated with higher odds of adherence relative to the reference category (OR = 23.50, 95% CI 2.10–263.55, *p* = 0.010); however, the wide confidence interval and a degenerate model intercept indicate partial separation, so only the direction of this association should be noted here. In summary, the significant predictors of adherence were older age, higher SCORE2 cardiovascular risk, higher HADS anxiety, current smoking, and self-employment. Discrimination was constrained by the class imbalance, and AUC was highest for the HADS anxiety model (0.801).

## 4. Discussion

According to ESC and ESH guidelines, effective BP regulation is based on a combination of pharmacological therapy and lifestyle modifications [5,6]. Despite the availability of effective therapeutic options, numerous studies have shown that a substantial proportion of patients did not achieve target BP values due to insufficient adherence to medication and lifestyle changes [7,8]. The results of this study suggest a relatively high level of self-reported adherence to antihypertensive medication. Among participants with controlled BP (<140/90 mmHg), 66.2% reported taking therapy in accordance with the physician’s instructions 100% of the time, whereas in the uncontrolled BP group this proportion was lower. This finding is in accordance with a recent ESC conclusion which suggests an association between higher adherence and better BP control [5]. Interestingly, nearly half of participants with uncontrolled BP reported 100% adherence to medication. This observation may be explained by the possible influence of social desirability bias during the self-assessment process [14,15]. Similar findings have been reported in other epidemiologic studies, including EUROASPIRE V, which have shown that self-assessed adherence can often overestimate actual adherence to prescribed pharmacotherapy [7,13,14,15]. Therefore, these relatively high-level rates of self-reported adherence observed in this study should be interpreted with caution, as self-reported measures are prone to social desirability and recall biases, potentially leading to an overestimation of actual medication-taking behaviour, particularly among participants with uncontrolled BP [14,15]. The failure to achieve optimal BP control despite self-reported 100% adherence to therapy can also indicate the need for improving the pharmacotherapy plan [14]. Furthermore, the discrepancy between high self-reported adherence and uncontrolled BP suggests that BP control depends on factors besides medication adherence alone. Even when patients take their medications as prescribed, BP may remain uncontrolled due to suboptimal pharmacotherapy intensity; therapeutic inertia; resistant hypertension; comorbidities such as obesity, diabetes, or chronic kidney disease; emotional stress; and unfavourable lifestyle characteristics (particularly excessive salt intake and physical inactivity) [16]. Therefore, uncontrolled BP in some participants who reported full adherence can reflect the multifactorial nature of hypertension management [5]. These findings highlight the importance of regular treatment review and strategies that address both pharmacological treatment and lifestyle modification. The COVID-19 pandemic illuminated the vulnerability of long-term hypertension management. Disruptions in healthcare access, reduced face-to-face consultations, and psychological stress were associated with poorer adherence to antihypertensive therapy and suboptimal BP control in many populations, emphasizing the importance of maintaining continuity of care during public health crises [17,18]. Previous studies have shown that better medication adherence is generally associated with improved health-related quality of life among patients with hypertension [19]. Patients managed in tertiary care facilities often have more severe disease, multiple comorbidities, and more complex treatment regimens, which may adversely affect quality of life despite intensive multidisciplinary management. In contrast, continuity of care and long-term patient–physician relationships, which are more characteristic of primary care settings in family medicine, may facilitate adherence and contribute to better health-related quality of life among patients with AH [20,21].

In this study, the most frequently used antihypertensive medication class was ACE inhibitors, followed by calcium channel blockers, diuretics and beta-blockers. The least frequently prescribed drug class was angiotensin receptor blockers (ARBs). The observed distribution is in accordance with most relevant European guidelines [5,6]. The reason why ARB use ranked lowest is due to prescribing restrictions by Croatian national insurance company policies (in effect until 2020). Self-reported 100% adherence across all antihypertensive drug classes ranged similarly. This similarity in adherence levels across different antihypertensive drug classes supports previous findings that adherence determinants are more closely linked to patient-related factors (personal attitudes towards illness and therapy, health literacy, patient–physician relationship, treatment motivation, and other psychosocial and socioeconomic factors) and less to intrinsic pharmacological properties of specific medications [22,23,24,25,26]. Psychological factors are increasingly recognized as important determinants of medication adherence among patients with AH, and the results in this study identified anxiety as the strongest predictor of poorer adherence. Although previous studies have focused mainly on depression, increasing evidence suggests that anxiety may also unfavourably impact long-term adherence and should be considered during hypertension treatment [11].

However, in addition to patient-related factors there are also age-related clinical determinants (often multimorbidity, polypharmacy, and age-related cognitive and functional decline) that influence adherence in older patients [27].

Recent consensus by ESC points to a need to improve adherence through simplified and individualized regimens, regular medication review with deprescribing when appropriate, and strengthened communication between physicians, patients, and caregivers. Continuity of care and patient-centred shared decision-making are key strategies associated with better adherence and improved long-term BP control in this population [28].

It should also be recognized that these patient-related factors are shaped by the broader social, cultural, and healthcare context. Previous studies have demonstrated substantial variation in adherence rates across countries and regions, reflecting differences in health literacy, socioeconomic status, beliefs about medications and illness, family and social support, accessibility of healthcare services and the quality of patient–physician communication [22,23,24,25,26,29]. In countries with universal health coverage, such as Croatia, financial barriers to medication access may be less pronounced than in some other countries’ healthcare systems; however, educational level, treatment beliefs, and motivation for long-term preventive therapy remain important determinants of adherence. In many African countries, inadequate medication adherence and difficulties in implementing lifestyle modifications remain major barriers to effective BP control. Limited access to healthcare services and medications, low health literacy, socioeconomic constraints, and cultural beliefs contribute to poor hypertension control, highlighting the importance of the above-mentioned social, cultural and national healthcare contexts [30,31]. Therefore, adherence patterns observed in Croatian primary care should be interpreted within their national and healthcare system contexts, as one setting may not necessarily be directly comparable to another. When examining BP control in relation to the antihypertensive drug class used, results showed that participants taking beta-blockers achieved better control compared to other medication classes. Since beta-blockers are not generally considered first-line therapy for uncomplicated AH, their use may indicate more intensive treatment strategies or the presence of specific clinical indications [5,6].

When comparing smokers with AH in Croatia to the overall EUROASPIRE V results [13], the prevalence of smokers is almost identical (18.6% in Croatia vs. 18.1% in all European countries). A direct comparison with data from more European countries presented in the article by Kotseva et al. [13] is limited by the fact that Croatian results are already embedded within the aggregate dataset. However, since Croatia accounts for only 7.4% of the total sample, its impact on the overall European estimates is almost negligible. It makes this comparison methodologically acceptable because its influence on the aggregate results does not significantly distort the comparative analysis. Despite high rates of counselling for smoking cessation in Croatia (86.2%) and across Europe (81.4%), a minority of patients actually quit smoking, both in Croatia (13.8%) and in other European countries (15.1%). These results may indicate that although patient counselling for smoking cessation is common, long-term smoking cessation remains low, suggesting that physicians’ advice without structured support is often insufficient for success. Partial smoking reduction among approximately one-quarter of smokers suggests a modest effect of counselling, but no comprehensive or long-term effect. Possible factors explaining such limited effectiveness of counselling include the fact that advice was primarily verbal and lacked structured follow-up support, a lack of systematic monitoring, and the influence of socioeconomic factors, addictive behaviours, and comorbidities [32,33,34,35]. In this study current smoking also emerged as a significant predictor of poorer adherence to antihypertensive therapy, consistent with previous findings relating smoking to suboptimal adherence among patients with AH [36].

Among 156 participants with AH, most participants reported receiving physician advice on salt intake reduction in the past three years. As many as 74.4% of participants reported that they have reduced salt intake in the past three years, which is similar to the European-level EUROASPIRE V survey (71.4%). An absence of counselling on salt reduction was significantly more common among women than men (26.6% vs. 11.7%; *p* = 0.03), and somewhat more frequent among participants aged ≥60 years, although this latter difference did not reach statistical significance (20.6% vs. 16.7%; *p* = 0.67). Several explanations are possible. Older patients may have been counselled less often if physicians assumed they had already received such advice previously. Women may have been advised less frequently because hypertension is still perceived, contrary to current epidemiological data, as a predominantly male disease, which can contribute to an underestimation of cardiovascular risk in women. These findings highlight the need for improved and more equitable communication and lifestyle counselling in these groups. The high percentage of respondents who reduced their salt intake may suggest that patients are aware of the importance of nutrition even beyond direct medical advice, which may be attributed to the influence of public health campaigns and improved general health literacy. For over a decade, systematic efforts to reduce excess salt intake have been carried out in Croatia and globally (e.g., the Croatian national CRASH campaign, the “Healthy Living” project, and campaigns such as World Salt Awareness Week), continuously raising awareness of hidden salt and thereby influencing behaviour change [37,38,39,40]. An interesting study from Japan analyzed Google Trends data and found that public interest in the term “salt reduction” increased dramatically, rising from 13.8% to 70.8% during the observed period from 2004 to 2021 [41].

The questionnaire results regarding physical activity suggest a lack of patient counselling. Just over half of the participants (58.3%) stated that they had not received medical advice to increase physical activity in the last three years, which is very similar to the aggregate EUROASPIRE V results, where 55.2% of participants reported a lack of counselling. The absence of professional advice was more frequent among women than men. A higher percentage of men (29.9%) reported an actual increase in physical activity compared to women (19.0%), which may suggest that men are more inclined towards physical activity or have a greater awareness of its importance [42]. More than half of the participants felt physically limited due to their illness, which likely results in predominantly light and mild physical activity engagement, which lacks cardioprotective effects. Namely, to achieve cardioprotective effects, regular moderate-to-intense aerobic activity is required [43,44]. Nevertheless, mild physical activity can lead to a gradual increase in intensity through additional education on the importance of progressing toward moderate and vigorous levels. The concept that “every minute counts” and a progress from light to more intense activity are part of the current recommendations by the WHO, ESC, and European Association of Preventive Cardiology (EAPC). Leading global and national health organizations and societies agree on the recommendation of a minimum of 150 min of moderate aerobic activity per week as the gold standard for health preservation [45,46,47,48,49,50,51,52]. Nearly one-third of the participants in this study (32.1%) reported that they do not exercise and have no plans to start within the next six months. This is very concerning given the established role of regular physical activity in reducing BP and cardiovascular risk. These results are comparable to the aggregate report of the primary arm of the EUROASPIRE V study, where nearly 40% of participants reported not performing any exercise and that they have no intention of starting any physical activity in the next 6 months [13]. This may indicate a low level of motivation for lifestyle changes, insufficient education regarding the benefits of physical activity, or the perception that medications alone are sufficient for treating AH [53,54]. Overall, both national and European EUROSPIRE V results regarding physical activity habits in patients with AH remain suboptimal, pointing to the need for additional interventions and the development of enhanced counselling strategies in this field. This is particularly worrying since it has been recently shown in the largest meta-analysis published until now that there is a significant inverse association between daily step count and all-cause mortality and CV mortality, with the more the better over the cut-off point of only 3867 steps/day for all-cause mortality and only 2337 steps for CV mortality [55]. Given the lack of time to devote to lifestyle habit counselling often faced by family physicians, involvement of final-year medical students in primary care could effectively bridge this gap. Their participation has shown clear benefits in AH screening and BP control [56]. Beyond this, medical students may contribute to preventive initiatives, including lifestyle counselling, cardiovascular risk education, and the promotion of healthy longevity.

This study has some limitations. Adherence to medication and lifestyle data were assessed only by self-reporting, which may lead to a social desirability bias or recall bias, as well as the under-reporting of unhealthy habits or the overestimation of positive behavioural changes. More objective methods (pharmacy refill data, electronic monitoring) combined with self-reporting would provide more precise data. These methods would enable a more accurate assessment of medication-taking behaviour over time and may help provide a more accurate estimate of adherence. Objective adherence measures ensure early identification of high-risk patients for poor BP control. Furthermore, generalizability is limited by selection bias, as only patients able to attend family medicine practices and willing to participate and patients with AH who reported current use of antihypertensive medication were included. Consequently, untreated individuals, those managed exclusively with lifestyle measures, or patients with limited access to healthcare were not represented. Therefore, the results should be interpreted as reflecting adherence patterns and BP control among treated patients with AH managed in family medicine practices rather than the entire population of patients with AH.

This study has several strengths. It was conducted according to the standardized, centrally coordinated EUROASPIRE V protocol, which ensured methodological rigour and allowed direct comparison of our findings with aggregate European data, and it was performed in the family medicine setting, where most patients with AH are actually managed, thereby enhancing the real-world applicability of the results.

## 5. Conclusions

The results of this study indicate that although patients generally self-report high adherence to therapy, a significant proportion of them still do not achieve optimal BP values. This issue is additionally associated with significant physical inactivity and smoking persistence. The effectiveness of smoking-cessation counselling and physical activity promotion appears to be limited, which highlights the need for a structured and systematic counselling framework. Identifying barriers to adherence, along with its continuous monitoring and the enhancement of lifestyle modifications, is essential for the comprehensive care of patients with AH. To achieve improved treatment outcomes, it is necessary to develop new forms of support strategies aimed at improving long-term persistence with both pharmacotherapy and lifestyle modifications.

## Figures and Tables

**Table 1:** Self-reported antihypertensive medication adherence in relation to BP control.

Self-Reported Adherence to Antihypertensive Medications				
N = 153	About half the time (50%)	Most of the time (75%)	Nearly all of the time (90%)	All of the time (100%)
BP <140/90 mmHg	1.5% N = 1	7.4% N = 5	25% N = 17	66.2% N = 45
BP ≥140/90 mmHg	2.4% N = 2	15.3% N = 13	32.9% N = 28	49.4% N = 42

BP—blood pressure.

**Table 2:** Proportion, full adherence, and BP control in relation to antihypertensive drug classes. Adherence was measured by self-reporting.

N = 156	ACE Inhibitors %	Beta-Blockers %	ARB %	Ca-Channel Blockers %	Diuretics %
Participants taking a specific antihypertensive drug class	64.7 (N = 101)	34.6 (N = 54)	17.3 (N = 27)	50.6 (N = 79)	43.6 (N = 68)
Self-reported adherence all of the time (100%)	54.5 (N = 55)	57.4 (N = 31)	59.3 (N = 16)	59.5 (N = 47)	58.8 (N = 40)
BP ≥ 140/90 mmHg	57.4 (N = 58)	40.7 (N = 22)	55.6 (N = 15)	60.8 (N = 48)	57.4 (N = 39)

ARB—angiotensin receptor blockers; ACE—angiotensin-converting enzyme; BP—blood pressure.

**Table 3:** Lifestyle characteristics and self-reported lifestyle changes in patients with AH.

		Gender			Age		
Lifestyle characteristics and changes in patients with AH	All N = 156 % (*n*)	Men N = 77 % (*n*)	Women N = 79 % (*n*)	*p* value	<60 years N = 54 % (*n*)	≥60 years N = 102 % (*n*)	*p* value
SMOKING							
Current smokers	18.6 (29)	19.5 (15)	17.7 (14)	0.83	27.8 (15)	13.7 (14)	0.05
Current smokers not having been offered professional advice to quit in past 3 years	13.8 (4)	20 (3)	7.1 (1)	0.6	13.3 (2)	14.3 (2)	1
Smokers—reduction in past 3 years	24.1 (7)	13.3 (2)	35.7 (5)	0.22	40 (6)	7.1 (1)	0.08
Smokers—abstinence in the past 3 years	13.8 (4)	20 (3)	7.1 (1)	0.60	13.3 (2)	14.3 (2)	1
SALT INTAKE							
Patients not having been offered professional advice to reduce salt intake in past 3 years	19.2 (30)	11.7 (9)	26.6 (21)	0.03	16.7 (9)	20.6 (21)	0.67
Patients who have reduced salt intake in the past 3 years	74.4 (116)	74 (57)	74.7 (59)	1	81.5 (44)	70.6 (72)	0.18
PHYSICAL ACTIVITY							
Patients not having been offered professional advice to increase physical activity in the past 3 years	58.3 (91)	50.6 (39)	65.8 (52)	0.07	53.7 (29)	60.8 (62)	0.40
Patients who have increased their physical activity in the past 3 years	24.4 (38)	29.9 (23)	19.0 (15)	0.15	31.5 (17)	20.6 (21)	0.34

## Data Availability

The data presented in this study are available from the corresponding author upon request.

## Supplementary Materials

The following supporting information can be downloaded at https://www.mdpi.com/article/10.3390/jcm15145376/s1, Table S1: Baseline characteristics of the study population (N = 156).

## Abbreviations

The following abbreviations are used in this manuscript:

AH	Arterial hypertension
BP	Blood pressure
WHO	World Health Organization
ESC	European Society of Cardiology
ESH	European Society of Hypertension
ACE	Angiotensin-converting enzyme
ARB	Angiotensin receptor blockers
EAPC	European Association of Preventive Cardiology
ASCVD	Atherosclerotic cardiovascular disease
HADS	Hospital Anxiety and Depression Scale
