# Supplementary material for: Medication Adherence and Lifestyle Changes Among Treated Patients with Arterial Hypertension in Family Medicine in Croatia According to the EUROASPIRE V Study: A Cross-Sectional Study
Source: J Clin Med. 2026 Jul 9;15(14):5376. doi: 10.3390/jcm15145376 (PMC13412628; doi:10.3390/jcm15145376)
Supplement: Supplementary file 1 [file jcm-15-05376-s001.zip › jcm-4372359-supplementary.pdf]

Table S1. Baseline characteristics of the study population (N = 156).

Characteristic	Overall (N = 156)	Missing, n
**Sex**		
Female	79 (50.6%)	
Male	77 (49.4%)	
**Age (years)**	65 (57, 72)	1
**Self-reported income level**		
Very low	1 (0.6%)	
Low	19 (12.2%)	
Middle	121 (77.6%)	
High	15 (9.6%)	
**Marital status**		
Married	114 (73.1%)	
Widowed	21 (13.5%)	
Never married	14 (9.0%)	
Divorced or separated	7 (4.5%)	
**Living arrangement**		
With somebody	128 (82.1%)	
Alone	28 (17.9%)	
**Highest level of education completed**		
Less than primary school	1 (0.6%)	
Primary school	9 (5.8%)	
Secondary school	63 (40.4%)	
High school	20 (12.8%)	
Technical training (post-secondary)	5 (3.2%)	
College or university	52 (33.3%)	
Postgraduate degree	5 (3.2%)	
Unknown	1 (0.6%)	
**Employment status**		
Retired	92 (59.0%)	
Full-time employed	44 (28.2%)	
Self-employed	9 (5.8%)	
Unemployed	5 (3.2%)	
Part-time employed	2 (1.3%)	

Characteristic	Overall (N = 156)	Missing, n
Sick leave	2 (1.3%)	
Unknown	2 (1.3%)	
**Ever smoked**		
Yes	56 (35.9%)	
No	100 (64.1%)	
**Current smoker** [a]		
Yes	29 (51.8%)	
No	27 (48.2%)	
**Smoking duration (years)** [b]	29 (20, 40)	
**Cigarettes per day** [b]	20 (10, 20)	
**Body mass index (kg/m^2)**	29.1 (26.4, 32.6)	
**Long-standing illness or disability**		
Yes	52 (33.3%)	
No	100 (64.1%)	
Don't know / unsure	4 (2.6%)	
**Frequency of regular physical activity**		
Never or rarely	84 (53.8%)	
Sometimes	52 (33.3%)	
Often	20 (12.8%)	
**Regular activity ≥30 min, 5 times/week**		
Yes	44 (28.2%)	
No	95 (60.9%)	
Don't know / unsure	17 (10.9%)	
**Told by a clinician of high blood cholesterol**		
Yes	97 (62.2%)	
No	52 (33.3%)	
Don't know / unsure	7 (4.5%)	
**Told by a clinician of diabetes**		
Yes, type 2	52 (33.3%)	
No	104 (66.7%)	
**HADS anxiety score**	6 (3, 9)	9
**HADS depression score**	4 (2, 8)	9
**Systolic blood pressure (mmHg)**	138 (128, 145)	

Characteristic	Overall (N = 156)	Missing, n
**Diastolic blood pressure (mmHg)**	82 (80, 90)	
**Heart rate (bpm)**	74 (68, 79)	18
**SCORE2 (%)**	11 (7, 17)	1
**Total cholesterol (mmol/L)**	5.24 (4.46, 6.06)	1
**HDL cholesterol (mmol/L)**	1.16 (1.02, 1.41)	1
**LDL cholesterol (mmol/L)**	3.09 (2.58, 3.90)	12
**Triglycerides (mmol/L)**	1.60 (1.19, 2.31)	1

Data are median (Q1, Q3) for continuous variables and n (%) for categorical variables; the number of participants with missing data is shown in the final column. Unless otherwise indicated, percentages are calculated using the full sample (N = 156).

[a] Percentages calculated among the 56 participants who had ever smoked.

[b] Smoking duration assessed among ever-smokers; cigarettes per day among current smokers.

bpm, beats per minute; HADS, Hospital Anxiety and Depression Scale; HDL, high-density lipoprotein; LDL, low-density lipoprotein; Q1/Q3, first/third quartile; SCORE2, Systematic Coronary Risk Evaluation 2.
